# Criminal behaviors and substance abuse in psychiatric patients

**DOI:** 10.1192/j.eurpsy.2023.1135

**Published:** 2023-07-19

**Authors:** B. Benatti, F. Achilli, S. Leo, L. Molteni, E. Piccoli, D. Gobbo, B. M. Dell’Osso

**Affiliations:** 1 Luigi Sacco University Hospital, Psychiatry 2 Unit; 2 CRC ‘Aldo Ravelli’ for Neuro-technology and Experimental Brain Therapeutics; 3 Luigi Sacco, Centro Psicosociale, Università degli Studi di Milano Statale, Milan, Italy; 4 Department of Psychiatry and Behavioral Sciences, Bipolar Disorders Clinic, Stanford University, Stanford, CA, United States

## Abstract

**Introduction:**

People with mental illness are overrepresented throughout the criminal justice system. In Italy, an ongoing process of deinstitutionalization has been enacted: the Judicial Psychiatric Hospitals are now on the edge of their closure in favor of small-scale therapeutic facilities (Residenze per l’Esecuzione delle Misure di Sicurezza - REMS). Law 81/2014 prescribes that a patient cannot stay in a REMS for a period longer than a prison sentence for the same index offense. Therefore, when patients end their duty for criminal behaviors, their clinical management moves back to outpatient psychiatric centers. Elevated risks of violent behavior are not equally shared across the spectrum of psychiatric disorders. In the past several years, multiple studies in the field of forensic psychiatry confirmed a close relationship between violent offenders and comorbid substance abuse.

**Objectives:**

In order to broaden the research in this area, we analyzed sociodemographic, clinical and forensic variables of a group of psychiatric patients with a history of criminal behaviors, attending an outpatient psychiatric service in Milan, with a focus on substance abuse.

**Methods:**

This is a cross-sectional single center study, conducted in 2020. Seventy-six subjects with a history of criminal behaviors aged 18 years or more and attending an outpatient psychiatric service were included. Demographic and clinical variables collected during clinical interviews with patients were retrospectively retrieved from patients’ medical records. Appropriate statistical analyses for categorical and continuous variables were conducted.

**Results:**

Data were available for 76 patients, 51,3% of them had lifetime substance abuse. Lifetime substance abuse was significantly more common in patients with long-acting injectable antipsychotics therapy, >3 psychiatric hospitalizations, history of previous crimes and economic crime (Table 1). Additionally, this last potential correlation was confirmed by logistic regression.

**Table 1.** 

	Lifetime substance abusers (N=39)		Non-lifetime substance abusers (N=37)	Proportion Difference	P-value		
	N	%		N	%		
Depot administration							
Yes	11	(28,9%)		0	(0%)	28,9%	0,02
Hospitalizations							
Four or more	25	(64,1%)		5	(33,3%)	30,8%	0,04
Economic crime							
Yes	15	(40,5%)		1	(6,7%)	33,8%	0,02
Previous crimes							
Yes	17	(51,4%)		2	(13,3%)	38,1%	0,02

**Conclusions:**

Data emerging from this survey provide new information about offenders in an Italian mental health service with a focus on lifetime substance abuse in these patients. Our preliminary results should be confirmed in larger sample sizes.

**Disclosure of Interest:**

None Declared

